# Bipolar Hemiarthroplasty With a Calcar Stem for the Management of a Failed Proximal Femoral Nail Anti-rotation Asia (PFNA2) in a Case of Geriatric Unstable Intertrochanteric Femur Fracture

**DOI:** 10.7759/cureus.65980

**Published:** 2024-08-01

**Authors:** Mukesh O Phalak, Tushar Chaudhari, Ajinkya K Chaudhari

**Affiliations:** 1 Department of Orthopaedics, Dr. D. Y. Patil Medical College, Hospital and Research Centre, Dr. D. Y. Patil Vidyapeeth (Deemed to be University), Pune, IND

**Keywords:** failed proximal femoral antirotation asia (pfna2), geriatric hip fracture, revision hip and knee surgery, unstable intertrochanteric fracture, bipolar hemi-arthroplasty, calcar replacement

## Abstract

Intertrochanteric (IT) femur fractures in the elderly population comprise a major part of geriatric trauma and fractures. There are various modalities of surgical management, ranging from intramedullary fixation and extramedullary fixation to even replacing the hip joint. Apart from the surgeon’s choice, other factors, such as geriatric age, bone quality and osteoporosis, medical comorbidities, life expectancy, pre-operative ambulatory status, muscle strength, type and pattern of fracture, and mental health of the patient, play vital roles in determining the ideal modality of management and the long-term outcome. The present case is a 75-year-old lady who had an IT fracture due to a domestic fall, managed surgically with a proximal femoral nail anti-rotation Asia (PFNA2) for an unstable fracture. She presented with blade back-out on the 11th day postoperatively. The patient was investigated thoroughly, and infection was ruled out. She was managed by the removal of the nail, followed by a cemented calcar-replacing bipolar hemiarthroplasty for an unstable comminuted IT fracture. The patient was ambulatory with a walker by the seventh postoperative day and without a walker by the sixth week, and she was self-sufficient in her activities of daily living. Every geriatric IT fracture must be evaluated thoroughly for contributing factors, such as osteoporosis and fracture pattern, to predict outcomes, and a tailor-made strategy of surgical management and stepwise physiotherapy must be provided to the patient for the best results.

## Introduction

Hip fractures are alarmingly common among the elderly age group, with intertrochanteric (IT) fractures constituting 50% of hip fractures in older individuals, over half of which are categorized as unstable [1]. IT fractures in the elderly present a frequent and daunting challenge for orthopedic surgeons. The Indian population shows a heightened vulnerability to osteoporosis and subsequent hip fractures [2]. With the increasing average lifespan and enhanced medical facilities, the incidence of these fractures has surged dramatically [3]. IT fractures affect activities of daily living due to impaired mobility and cause problems with balance [4]. Osteoporosis is a major contributor to the fracture parameters of IT femur fractures, such as comminution and stability, and its significance is greater than previously understood [5]. The main aim of managing IT femur fractures is to rehabilitate the patient to a self-sufficient state and allow them to engage in activities of daily living effortlessly. There is a variety of fixation methods available - an intramedullary type of fixation by nailing and replacement options, as well as extramedullary options like a dynamic hip screw (DHS) and a side-plate assembly.

The debate on the gold standard of treatment is constant. Factors like patients’ medical conditions and comorbidities, bone quality and strength, pre-fracture ambulatory status, fracture pattern, and comminution decide an appropriate plan of management for elderly patients with IT femur fractures [6]. Conventionally, stable IT fractures are generally treated with the compression hip screw [7]. However, this screw type has a higher likelihood of cut-out failure (6-19%) in cases involving unstable IT fractures [8,9]. Recently, several orthopedic surgeons have started recommending the newer proximal femoral nail anti-rotation Asia (PFNA2) implant over the DHS for treating stable IT fractures in older patients. The advantages of PFNA2 include faster procedures, smaller incisions, intramedullary fixation, and the ability to achieve compression at the fracture site. An inferior screw placement in PFNA2, with an appropriate tip-apex distance, reduces the risk of screw cut-out [10]. There are successful outcomes after partial or total hip replacement (THR) in cases with unstable multi-fragmentary IT femur fractures. The advantages of this modality include early mobilization, reducing the risk of prolonged bed rest and associated complications, shorter hospital stays, and improved patient satisfaction. We present a case of failed PFNA2 with blade cut-out managed with bipolar hemiarthroplasty using the calcar substitution design as a primary treatment for unstable osteoporotic IT fractures.

## Case presentation

**Figure 1:**
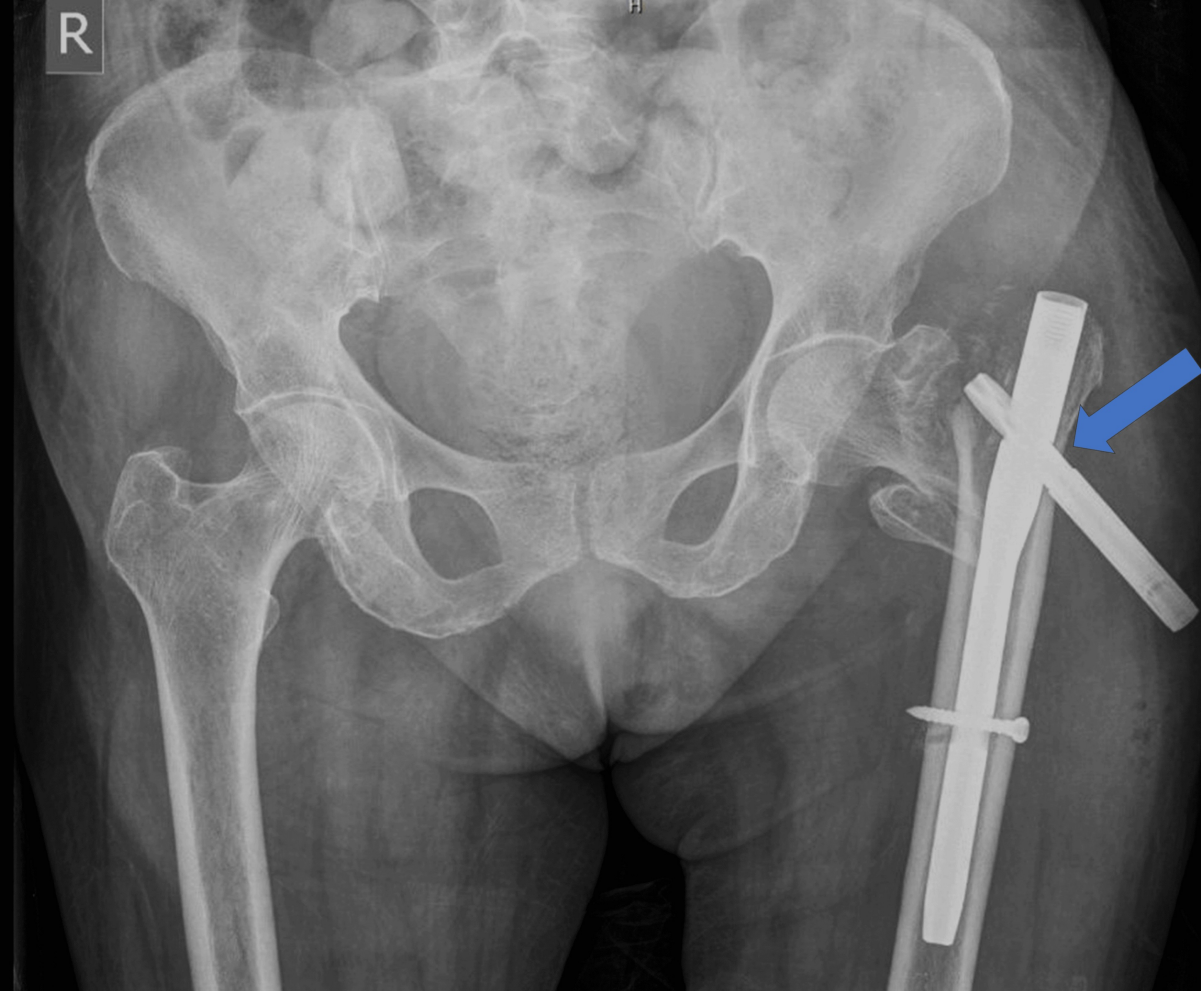
Failed PFNA2 The blue arrow in the PBH radiograph shows blade back-out and implant failure on the left side. PBH: Pelvis with both hips; PFNA2: Proximal femoral nail anti-rotation Asia

We present the case of a 75-year-old female who came to us with complaints of excruciating pain in the left hip and a history of constant soakage from the operative site. The patient had suffered a fall two weeks prior and was diagnosed with a left IT femur fracture. She underwent closed reduction and internal fixation with a PFNA2. Postoperatively, the patient was initiated on bedside sitting on the second postoperative day and non-weight-bearing walking from day 5. From the seventh postoperative day, the patient experienced persistent soakage from the surgical site, necessitating dressing changes twice daily. She presented to us on the 11th postoperative day with intense pain in the left hip and a soaked dressing. The attendants with the patient provided a history of two to three instances of weight bearing on the operated side, despite a warning from the then-treating orthopedic surgeon. Clinically, the orthopedic surgeons suspected a surgical site infection. A fresh radiograph of the pelvis with both hips (PBH) revealed a blade back-out and PFNA2 implant failure (Figure 1).

**Figure 2:**
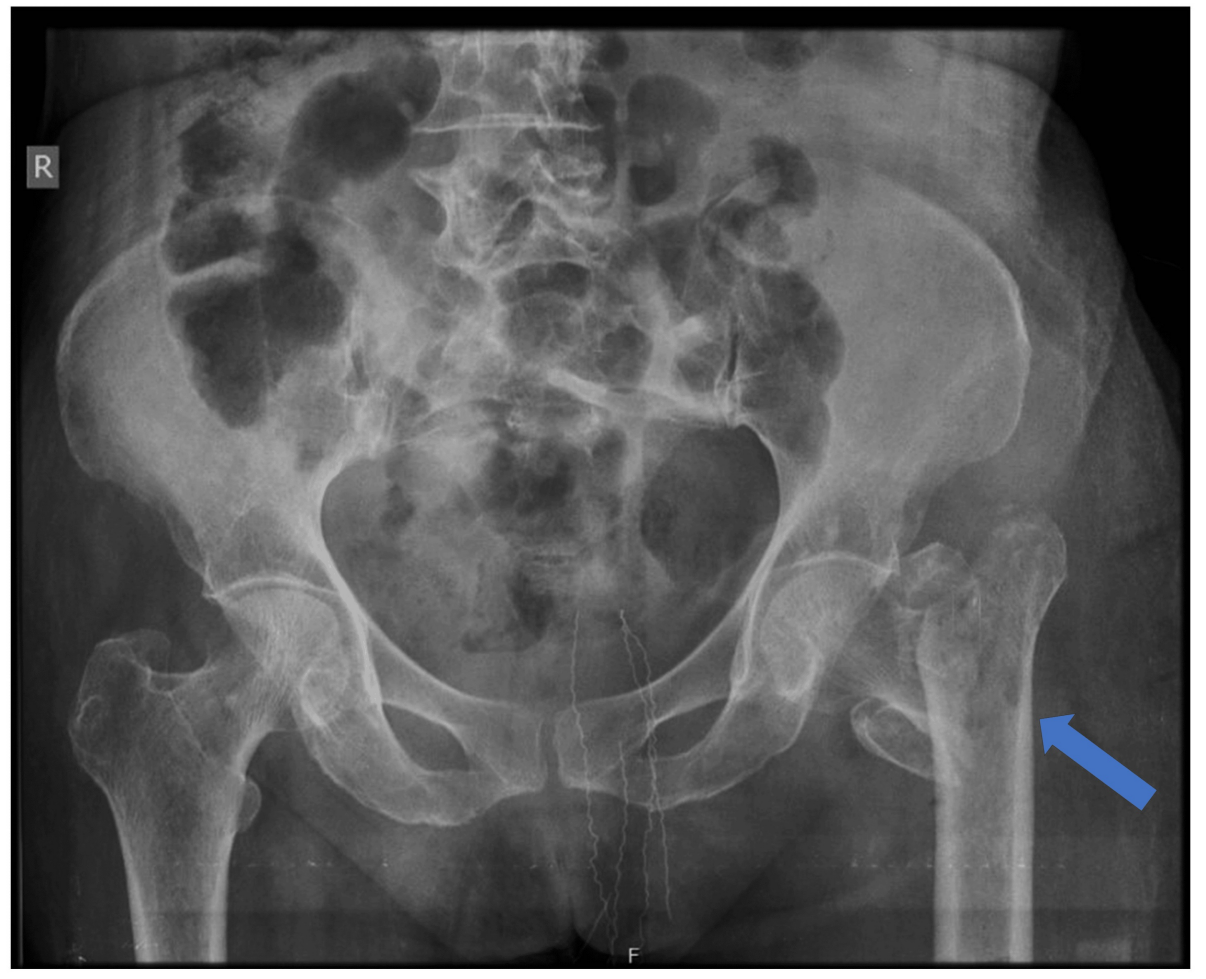
X-ray of PBH after the removal of the PFNA2 The blue arrow shows a comminuted unstable intertrochanteric femur fracture. PFNA2: Proximal femoral nail anti-rotation Asia; PBH: Pelvis with both hips

Laboratory investigations were conducted to rule out infection. The total leucocyte count (TLC = 6500/µL, normal range: 4000-10,000 µL), C-reactive protein (CRP = 6.55 mg/dL, normal < 5.0 mg/dL), and erythrocyte sedimentation rate (ESR = 22 mm/hr, normal up to 30 mm/hr in females over 50 years of age) were all within near-normal limits, showing no significant signs of infection. As a confirmatory test, a swab was sent for culture studies, which tested "no growth" of any microbe. Given that this was a second surgery following an implant failure in the initial surgery, a step-wise approach was planned. In the second surgery, all hardware was removed from the patient’s left hip, local tissue was debrided until fresh, bleeding, healthy tissue was obtained, and a thorough wash with 8 L of normal saline using a pulse lavage was administered. The patient received postoperative antibiotic cover with intravenous cefuroxime and amikacin for eight days and five days, respectively. A repeat titer of ESR, CRP, and TLC was done on the fifth postoperative day, showing a decreasing trend: ESR = 15 mm/hr, CRP = 3.22 mg/dL, and TLC = 4500/µL. The radiograph of PBH was repeated to study the fracture pattern and comminution (Figure 2).

**Figure 3:**
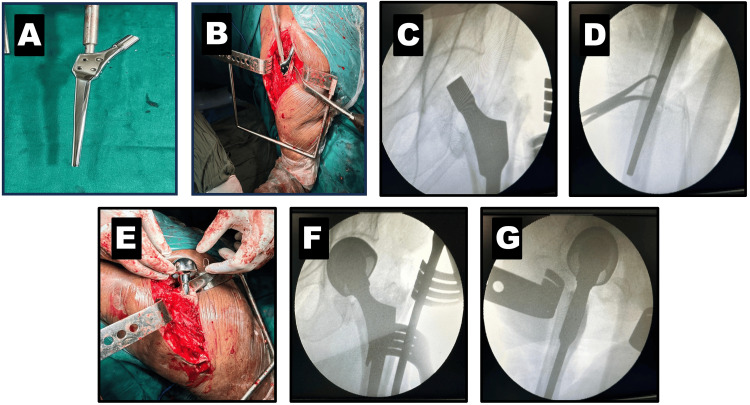
Intraoperative images of calcar replacing bipolar hemiarthroplasty A) Calcar replacing stem design; B) Cemented calcar stem maintaining version; C) C-arm image of AP view of calcar stem; D) C-arm image of lateral end of calcar stem; E) Implanted head before reduction of the hip; F) C-arm image AP view after final implantation; G) C-arm image of lateral view after final implantation

**Figure 4:**
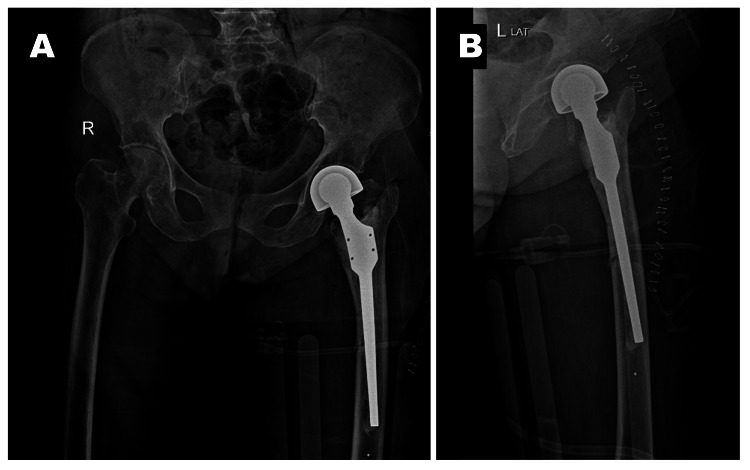
Post-operative X-ray of calcar replacing bipolar hemiarthroplasty A) Anteroposterior view of X-ray PBH; B) Lateral view of X-ray PBH The PBH radiograph shows bipolar hemiarthroplasty of the left side with cemented calcar replacing the stem. PBH: Pelvis with both hips

Given the implant failure in the initial fixation attempt and the complex fracture pattern classified as Boyd and Griffith type III, a calcar-replacing stem of the prosthesis was deemed the best choice. The third surgery involved a calcar-replacing cemented bipolar hemiarthroplasty of the left hip (Figure 3). The patient was operated on under spinal and epidural anesthesia in the lateral decubitus position, using the posterior Southern Moore approach to the hip. The external rotators were tagged with a 1-0 vicryl and detached from their insertion to free the femur. The head and neck were extracted with a corkscrew, and a small posterior fragment was also removed. The box cut was made comparatively shallow in our case to avoid splitting the lateral wall, considering the fracture involving the lesser trochanter. The canal finder was used to prepare the femur, followed by broaching and the standard steps of bipolar hemiarthroplasty. The trial calcar stem was inserted to confirm the size, varus-valgus alignment, and allowable sink of the calcar region of the prosthesis for the best fit and results. The sink was measured under traction to approximate the final length and compare it with the opposite length for a zero offset head. The calcar-replacing stem was cemented into the proximal femur, maintaining the version and level of the sink as per the trial placement. After cementing the calcar stem, the trial head was used to assess the final offset and tested by reducing the hip joint. We checked the stability of the prosthesis with the passive range of motion, shuck test, and limb length intraoperatively before the final implantation. The final implants were then inserted, and the hip joint was reduced. The passive range of motion, shuck test, and limb length were re-assessed, and the outcome was as per the surgeons’ satisfaction. The external rotators were sutured back using the Ranawat technique, and closure was completed. The step-by-step approach, constant assessment, and intraoperative C-arm guidance were helpful for a complicated revision surgery like this one, which helped the surgeon to expect a good outcome despite contradicting factors such as geriatric age, poor bone quality and muscle strength, revision surgery, complex fracture pattern with comminution, and high risk for implant subsidence. The postoperative radiographs are shown in Figure 4.

Postoperatively, the patient received intravenous antibiotics for five days, comprising intravenous cefuroxime and intravenous amikacin, as well as oral antibiotic cover with a fixed-dose combination of cefuroxime and clavulanic acid until suture removal to minimize the risk of infection and further surgeries. The patient was gradually mobilized using physiotherapy. The first two days postoperatively included chest physiotherapy, incentive spirometry, ankle pumps, and log-rolling exercises. An abduction pillow support was used to prevent dislocation of the hip, especially at night. The patient was allowed to sit in bed on the second day and stand with the support of a walker on the fifth postoperative day after the pain subsided as per the patient's comfort. The patient began non-weight-bearing walking from the seventh postoperative day until three weeks postoperatively and gradually started weight-bearing. The patient commenced complete weight-bearing by the sixth week postoperatively and resumed daily activities independently. The patient was assessed at the fourth, sixth, and eighth weeks postoperatively for limb length discrepancy and using the Harris Hip Score. There was a limb length discrepancy of 1.5 cm postoperatively, which was accepted considering the challenges faced in the case. The Harris Hip Score was 56 in the fourth week, 60 in the sixth week, and 74 in the eighth week, indicating a good prognosis for a complicated case.

## Discussion

Osteoporosis structurally weakens the bones and leads to an increased risk of fractures, such as IT fractures, which become challenging to manage. The associated medical issues - comminution, osteoporosis, and instability - often hinder early full weight bearing, a crucial factor leading to unsatisfactory outcomes [11,12]. The most common complication of surgery for IT fractures is the failure of the fixation in the femoral head and neck, which requires the reinsertion of a nail or replacement of the hip joint. Despite the potential advantages of the proximal femoral nail (PFN) for treating unstable fractures in the elderly, problems such as screw cut-out and fracture fixation failure are common in this age group. These complications arise due to poor purchase in the bone and reduced mechanical stability due to impaired bone quality, leading to biomechanical failure [13]. Weight-bearing is a stressor in elderly patients and may result in concentrated pressure on the distal intramedullary nail, locking nail, and nail junction, thus causing long-term stress on these fracture areas and adding to the risk of periprosthetic fracture. The prevalence of issues and failures following surgery varies depending on the adoption of fixation techniques, screw placement, and postoperative protocols.

Implant failure is a known disadvantage of PFN in osteoporotic bone. Imprecise placement and inadequate depth of the spiral blade, as well as the quality of the bone, are critical factors that could lead to the severing of the head and neck of the screw. There is no fixation device that can successfully produce a stable internal fixation and promote early activity in patients with severe osteoporosis, which is mostly associated with unstable IT femur fractures. Fan et al. demonstrated good outcomes with bipolar hemiarthroplasty for IT femur fractures as compared with total hip arthroplasty [14]. According to research by Bonnaire et al., the lack of success in surgical techniques such as DHS, gamma nails, and PFN postoperative cutting is overwhelmingly linked to osteoporosis; in their study, they report a substantial correlation between a lower bone mineral density (i.e., <0.6 g/cm³) and an increased risk of fixation failure [15].

The main concerns with geriatric patients are bone quality, decreased muscle strength, and a complicated unstable fracture, which makes fixation challenging for the surgeon and delays weight bearing [16]. The non-ambulatory status adds to the risk of complications and deterioration of the patients' condition. On the other hand, if replacement is prioritized over fixation for such cases, there are some significant advantages, such as immediate ambulation, less risk of implant failure, and a mobile and painless joint. The available options following a failed PFN procedure include refixation with an intramedullary nail, bipolar hemiarthroplasty, and THR. In the present case, bipolar hemiarthroplasty was chosen owing to its lower rate of dislocation with bipolar compared to THR in cases of traumatic proximal femoral fractures [17]. The matter of concern was whether to replace the calcar femoral, which, in many of these unstable IT fractures, is deficient. The strength of the calcar is crucial in deciding the stability and long-term outcome of the replacement surgery and affects limb length, which is an important clinical factor. We used a cemented calcar-replacing bipolar hemiarthroplasty, which replaces the deficient area in the calcar zone, yielding immediate stability without fear of subsidence. Although there was a certain risk of mortality because of cementation, an uncemented implant, when used in osteoporotic bone, may carry the risk of periprosthetic fracture, subsidence, and reduced stability compared to a cemented type of implant.

In unstable fractures like these, calcar-replacing bipolar hemiarthroplasty offers significant advantages, such as rigid fixation, early ambulation, a lower risk of failure compared to PFNA2, and low complication rates [18]. The results of replacement are promising; however, there are certain drawbacks, such as increased blood loss, risk of dislocation, and infection when compared to fixation. Kim et al. compared the calcar-replacement prosthesis with nailing in a sample size of 29 patients and found equally favorable outcomes in both groups, with a cut-out rate of 7% [19]. A prime limitation of this case report is the singularity of the case; a study with a larger sample size to determine the best modality following a failed PFNA2 is warranted. However, finding the single best surgical treatment is difficult, owing to multiple factors playing a key role in the outcome of IT femur fractures, particularly in the elderly. Thus, every case must be evaluated by the surgeon, and case-based tailored management must be provided to the patient, along with proper counseling on the pros and cons of the modality of management for IT femur fractures.

## Conclusions

The main factors contributing to the biomechanical failure of internal fixation are improper implant placement, inadequate tip-apex distance, pronounced osteoporosis, and incorrect positioning of the helical blade. The degree of osteoporosis is closely correlated with the occurrence of internal fixation failure. If internal fixation is unsuccessful, it is recommended to consider either replacing the joint with an artificial one or re-fixing it. We demonstrated a good outcome with cemented calcar-replacing bipolar hemiarthroplasty in an elderly female whose index surgery of internal fixation with PFNA2 had failed due to blade cut-out. We recommend tailoring the therapy based on the patient’s age, level of physical activity, and bone density. A calcar-replacement hemiarthroplasty in cases of failure is a good option, offering quick recovery, less risk of mechanical failure, and a stable, pain-free, functional joint.
